# A rare case of human taeniasis caused by *Taenia saginata* with species undetermined cysticercosis

**DOI:** 10.1017/S003118202200169X

**Published:** 2023-03

**Authors:** Jie Hou, Weilin Chen, Rong Chen, Chunlei He, Ying Ma, Junyan Qu

**Affiliations:** 1 Department of Laboratory Medicine, West China Hospital, Sichuan University, Chengdu, Sichuan, China; 2 Department of Clinical Laboratory, The First People's Hospital of Shuangliu District, Chengdu/West China (Airport) Hospital, Sichuan University, Chengdu, Sichuan, China; 3 West China School of Medicine, Sichuan University, Chengdu, Sichuan, China; 4 Center of Infectious Disease, West China Hospital, Sichuan University, Chengdu, China

**Keywords:** Human cysticercosis, *Taenia asiatica*, *Taenia saginata*, *Taenia solium*, taeniasis

## Abstract

Taeniasis and cysticercosis, which are caused by *Taenia saginata*, *Taenia solium* and *Taenia asiatica*, are zoonotic parasitic infections with a significant disease burden worldwide. There is consensus amongst experts that *T. saginata* is a common tapeworm that causes taeniasis in humans as opposed to cysticercosis. This case study of a middle-aged Tibetan man conducted in 2021 challenges the prevailing notion that *T. saginata* exclusively causes taeniasis and not cysticercosis by documenting symptoms and laboratory studies related to both taeniasis and multiple cysticercosis. The patient's medical record with the symptoms of taeniasis and cysticercosis was reviewed, and the tapeworm's proglottids and cyst were identified from the patient by morphological evaluation, DNA amplification and sequencing. The patient frequently experienced severe headaches and vomiting. Both routine blood screenings and testing for antibodies against the most common parasites were normal. After anthelmintic treatment, an adult tapeworm was found in feces, and medical imaging examinations suggested multiple focal nodules in the brain and muscles of the patient. The morphological and molecular diagnosis of the proglottids revealed the Cestoda was *T. saginata.* Despite the challenges presented by the cyst's morphology, the molecular analysis suggested that it was most likely *T. saginata*. This case study suggests that *T. saginata* infection in humans has the potential to cause human cysticercosis. However, such a conclusion needs to be vetted by accurate genome-wide analysis in patients with *T. saginata* taeniasis associated with cysts. Such studies shall provide new insights into the pathogenicity of *T. saginata*.

## Graphical abstract

**Figure:**
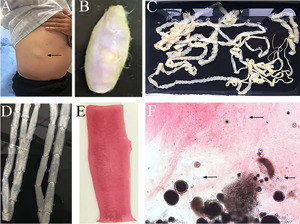

## Introduction

The zoonotic infection taeniasis is most commonly caused by *Taenia saginata*, *Taenia solium* and *Taenia asiatica*, which are associated with ingesting raw or undercooked, infected beef meat, pork meat or pork viscera, respectively (Ito *et al*., 2003; Eichenberger *et al*., 2020). The life cycle of pathogenic *Taenia* species involves humans as definitive hosts, who harbour the adult stage in their small intestines. Cattle (*T. saginata*) or pigs (*T. solium* and *T. asiatica*) can act as intermediate hosts, who hold the larval stages as cysts that dwell themselves under the skin and in the muscles, nervous system or visceral organs such as the liver of the hosts (Qian *et al*., 2020). Tapeworm eggs or gravid proglottids are passed in feces, and the eggs can survive for days to months in the environment (Jansen *et al*., 2021). Humans can also serve as accidental intermediate hosts when exposed to embryonated eggs (this role is normally fulfilled by *T. solium*) released by themselves (autoinfestation) or by another tapeworm carrier living in close contact with the subject or involved in contaminated food and water, causing infection in various organs of the human body with the larvae also known as cysticerci (human cysticercosis) (Garcia *et al*., 2003; Garcia, 2018; Alroy and Gilman, 2020). While *T. saginata* is responsible for a significant number of cases of taeniasis in humans, it is not thought to be the aetiological agent of cysticercosis in humans because they do not appear to be an accidental intermediate host for this parasite (Garcia *et al*., 2020). It is not known whether *T. asiatica* causes cysticercosis in humans (Eom *et al*., 2020).

Twelve cases of *T. saginata* cysticercosis in humans had been described in the literature before 1972, and these diagnoses were based on autopsy and morphological examination by hookless scoleces (De Rivas, 1937; Tanasescu and Repciuc, 1939; Asenjo and Bustamente, 1950; Niiio, 1950; Bacigalupo, 1956; Abuladze, 1964; Goldsmid, 1966; Pawlowski and Schultz, 1972). In addition, *T. saginata* infection in adults can occur simultaneously with human cysticercosis (Pawlowski and Schultz, 1972). The infection of humans with the larvae (cysticerci) of *T. saginata* appears to be an exceptional situation (Pawlowski and Schultz, 1972). Medical imaging testing, clinical/exposure criteria, serologic testing, histological pathology and molecular identification all help in the diagnosis of human cysticercosis (Del Brutto *et al*., 2017). Due to the lack of molecular methods readily available in most hospital settings, proper species identification in all reported cases of human cysticercosis is impossible.

Numerous molecular procedures have been developed to identify *Taenia* spp. responsible for human taeniasis, using mitochondrial and ribosomal DNA, repetitive DNA sequences and/or genes encoding relevant antigens as specimens (Flores *et al*., 2018). The *cytochrome c oxidase I* (*COX1*) gene is a widely used DNA barcoding marker because the rapid rate of evolution allows for the differentiation of not just closely related species, such as the *Taenia* genus, but also phylogeographic groups within a single species (Hebert *et al*., 2003; Galimberti *et al*., 2012; Zhang *et al*., 2014). Evidence suggests the HDP2 segment is a ribosomal DNA sequence useful for species-specific molecular diagnosis of human intestinal taeniasis (Gonzalez *et al*., 2000; Flores *et al*., 2018). In this case study, we identified the case of a middle-aged Tibetan man with taeniasis owing to *T. saginata* and cysticercosis that had impacted his brain and muscles diagnosed by morphological and molecular methods. We also used *COX1* to create a phylogenetic tree of *Taenia*.

## Materials and methods

### Clinical examinations

The electronic medical records of the study subject were retrospectively reviewed, and the following demographic and clinical information was collected: age, gender, past medical history, physical examinations, diagnosis, underlying conditions, drug use, iatrogenic procedures, laboratory tests, computed tomography (CT), enhanced magnetic resonance imaging (MRI), ultrasound scan and clinical outcomes.

### Morphological evaluation

Two obvious cysts were detected in the muscles under the right anterior thoracic wall and right midaxillary line through palpation and ultrasound scan, respectively. The cyst biopsy of the right midaxillary line and the proglottids from the patient after deworming treatment was collected and observed morphologically. The gravid proglottids and cystic lesions were fixed in a 10% formalin solution, stained with the hydrochloric acid-carmine solutions and observed under a microscope for pathological diagnosis.

### Amplification and sequencing

A portion of the cyst isolated from the right midaxillary line and proglottids was stored in 70% ethanol at −20°C until subsequent molecular diagnosis. Total genomic DNA was extracted by using the *Trelief*™ Animal Genomic DNA Kit (Tsingke Biotechnology, Beijing, China) in accordance with the manufacturer's instructions. Mitochondrial DNA was analysed by polymerase chain reaction (PCR) targeting *COX1* using species-specific forward primers Tsag_COX1/F for *T. saginata* (positions 322–348) and a common reverse primer Tae_COX1/R for *Taenia* (positions 1148–1129) (Yamasaki *et al*., 2004), cestode forward primers JB3 (positions 1–24) and reverse primers JB4.5 (positions 444–421) (Bowles *et al*., 1992; Bowles and McManus, 1994; Tappe *et al*., 2016). The HDP2 fragment was amplified with the forward primer PTs7S35F1 and the reverse primer PTs7S35R1 (Gonzalez *et al*., 2000). Details of the PCR primers and the reaction conditions are provided in Table 1. PCRs were run in a 30-*μ*L reaction mixture composed of 15-*μ*L I-5™ 2× High-Fidelity Master Mix (Tsingke Biotechnology), 1-*μ*L DNA template, 10 pmol of each primer and 12-*μ*L double-distilled water. The thermocycler parameters of the PCR amplification were as follows: 98°C for 3 min; 39 cycles composed of denaturation at 98°C for 10 s, annealing according to the primers and elongation at 72°C for 15 s. After 39 cycles, the reaction was terminated with an elongation step at 72°C for 5 min, followed by a final hold at 4°C. The amplicons were observed using a 1.5% agarose gel under ultraviolet light and then purified, after which Sanger sequencing methods were carried out at Tsingke Biotechnology or Sangon Biotech (Shanghai, China). The sequenced amplicons were compared to the GenBank database entries using NCBI BLAST (Johnson *et al*., 2008), wherein the organism was limited to *Taenia* (taxid: 6202) and the query coverage was 100%.

**Table 1.** Primer pairs used for PCRs

Order	Target	Primer names	Orientation	Positions	Sequences (5′–3′)	PCR products (bp)	Annealing	Samples	BLAST per cent identity^a^
1	COX1	Tsag_COX1/F	Forward	322–348	TTGATTCCTTCGATGGCTTTTCTTTTG	827	60°C for 30 s	Proglottids cyst^b^	*Taenia saginata* 99.27–100% *Taenia asiatica* 96.25–96.37%
		Tae_COX1/R	Backward	1148 –1129	GACATAACATAATGAAAATG				
2	COX1	JB3	Forward	1–24	TTTTTTGGGCATCCTGAGGTTTAT	444	51°C for 10 s	Proglottids cyst^c^	*T. saginata* 99.24–100%
		JB4.5	Backward	444–421	TAAAGAAAGAACATAATGAAAATG				
3	HDP2	PTs7S35F1	Forward	1–22	CAGTGGCATAGCAGAGGAGGAA	599	57°C for 25 s	Cyst	*T. saginata* 99.67–99.83% *T. asiatica* 95.66–99.67% *Taenia solium* 92.99–97.33%
		PTs7S35R1	Backward	599–577	GGACGAAGAATGGAGTTGAAGGT				

a The per cent identity was obtained from the NCBI BLAST webpage and the organism was limited to the *Taenia* (taxid: 6202) genus and the query coverage was 100%.

b Only proglottids were successfully amplified.

c Both proglottids and cysts were successfully amplified.

### Phylogenetic analysis

Multiple sequence alignment was constructed with Clustal W, Clustal Omega and SnapGene software. Phylogenetic analysis of the sequences of COX1 identified in this study and the reference sequences of *Taenia* species available in the GenBank database (Table 2) were constructed using the maximum-likelihood method by MAGA X. The bootstrap values were calculated with 2000 replications, and the nucleotide substitution of the Tamura–Nei model was adapted.

**Table 2.** Reference COX1 sequences of *Taenia* species in GenBank

*Taenia* species	GenBank accession no.
*T. asiatica*	NC_004826.2
*T. saginata*	NC_009938.1
*T. solium*	NC_004022.1
*Taenia hydatigena*	NC_012896.1
*Taenia multiceps*	NC_012894.1
*Taenia pisiformis*	NC_013844.1
*Taenia crassiceps*	NC_002547.1
*Taenia arctos*	NC_024590.1
*Taenia crocutae*	NC_024591.1
*Taenia regis*	NC_024589.1
*Taenia laticollis*	NC_021140.1
*Taenia madoquae*	NC_021139.1
*Taenia martis*	NC_020153.1
*Taenia ovis*	NC_021138.1
*Taenia serialis*	NC_021457.1
*Taenia twitchelli*	NC_021093.1

## Results

### Case presentation

A middle-aged Tibetan man from China was admitted to our hospital with a 7-year history of recurring severe headaches and vomiting. The headache had started with vomiting, nausea, dizziness and chest tightness, especially on the right side, without any apparent predisposing cause. Weekly, he had 2 or 3 episodes of non-projectile vomiting without bile or coffee-like particles, and the headaches lasted for nearly a day each time. There were no other symptoms, such as fever, chills, convulsions, seizures, increased intracranial pressure or other discomforts. He had not been treated for these signs and symptoms before admitting to our hospital in December 2020. After being fully diagnosed in our hospital, his comorbidities included hypertension, fatty liver, liver insufficiency, paranasal sinusitis and intestinal bacterial infection. The immunosuppression was not found. Additionally, he spent a significant amount of time in a pastoral area that is an epidemic province for taeniasis, although no tapeworm infections were diagnosed before his admission to our hospital. He used to ingest raw beef, which might have led to the ingestion of the cysticerci of *T. saginata*. Moreover, as far as pork consumption is concerned, his family's eating habit, like most Han Chinese, were eating it cooked, ruling out the possibility of related parasites.

**Fig. 1.**
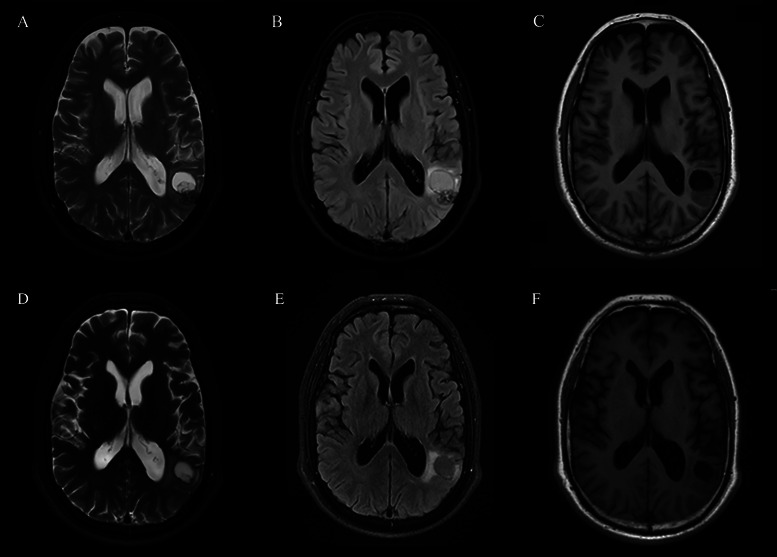
MRI shows that the largest cystic nodule was located next to the left ventricular triangle: (A–C) half a month before antiparasitic treatment and (D–F) half a month after antiparasitic treatment.

**Fig. 2.**
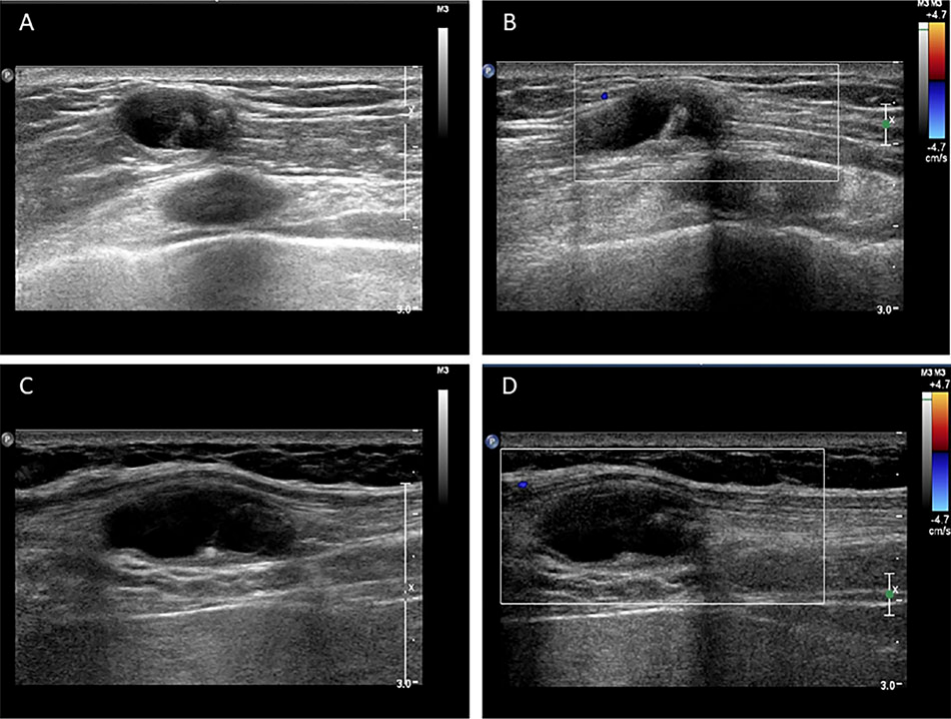
Ultrasound scan shows hypoechoic mass containing cysticercus with calcification in the muscles of (A, B) right anterior thoracic wall (19 × 8 × 15 mm^3^) and (C, D) right midaxillary line (26 × 10 × 19 mm^3^).

**Fig. 3.**
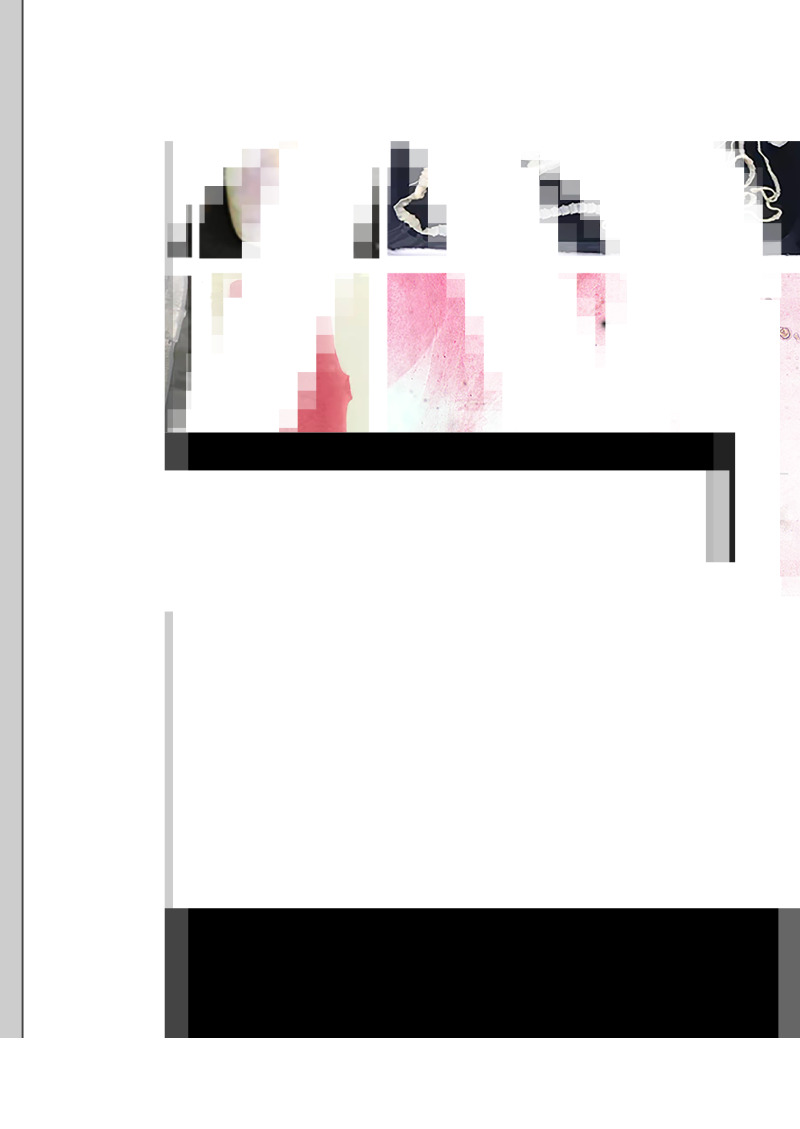
Tapeworm materials from the patient: (A) subcutaneous mass; (B) cyst; (C) adult tapeworm; (D) unstained proglottids; (E) a stained proglottid and (F) cystic lesion with hydrochloric acid-carmine staining showing presumed deciduous hooks (black arrows).

The routine laboratory blood tests, including eosinophil and lymphocyte count, were completely normal apart from the elevated inflammation markers (C-reactive protein and neutrophil count). *Schistosomes*, *Clonorchis sinensis*, *Echinococcus granulosus* and *Toxoplasma gondii* antibodies were negative in his serum, as were *Cryptococcus* capsular antigen and next-generation sequencing results from cerebrospinal fluid. Multiple intracranial nodules affecting the supratentorial and infratentorial cerebral parenchyma were shown in detail on CT and MRI of the head, indicating possible intracranial parasitic infection (Fig. 1). Ultrasound scan confirmed the presence of 2 palpable and soft masses located in muscles, which were approximately 19 × 8 × 15 mm^3^ under the right chest wall and 26 × 10 × 19 mm^3^ under the right midaxillary line (Fig. 2). A subsequent biopsy of the mass (Fig. 3) showed larval-like tissue, peripheral fibrous tissue hyperplasia, lymphocytic infiltration and hyaline degeneration. The patient was probably diagnosed with taeniasis and cysticercosis and treated with oral albendazole (400 mg, twice daily) over 2 weeks. Hydrocortisone 50 mg was provided 2 days after the first albendazole treatment to counteract any potential negative effects on the central nervous system. After only 2 days of this antiparasitic treatment, the adult tapeworm was eliminated through the patient's feces (Fig. 3). When compared with the first MRI (half a month before antiparasitic treatment), the second MRI (half a month after antiparasitic treatment) demonstrated a slightly smaller focus (Fig. 1). The headache and vomiting resolved, and the patient remained symptom free over a 3-month follow-up period.

### Morphological observation

The adult tapeworm was milky white, flat and long like a belt, thin and translucent, and the main segments are shown in Fig. 3. The uterine branches in the gravid proglottids were regular, and each side had approximately 15–30 branches (Fig. 3). The slide of the cyst showed no obvious typical features; however, some presumed deciduous hooks were found in the same slide, leading to indistinguishable morphology (Fig. 3).

### Molecular identification

**Fig. 4.**
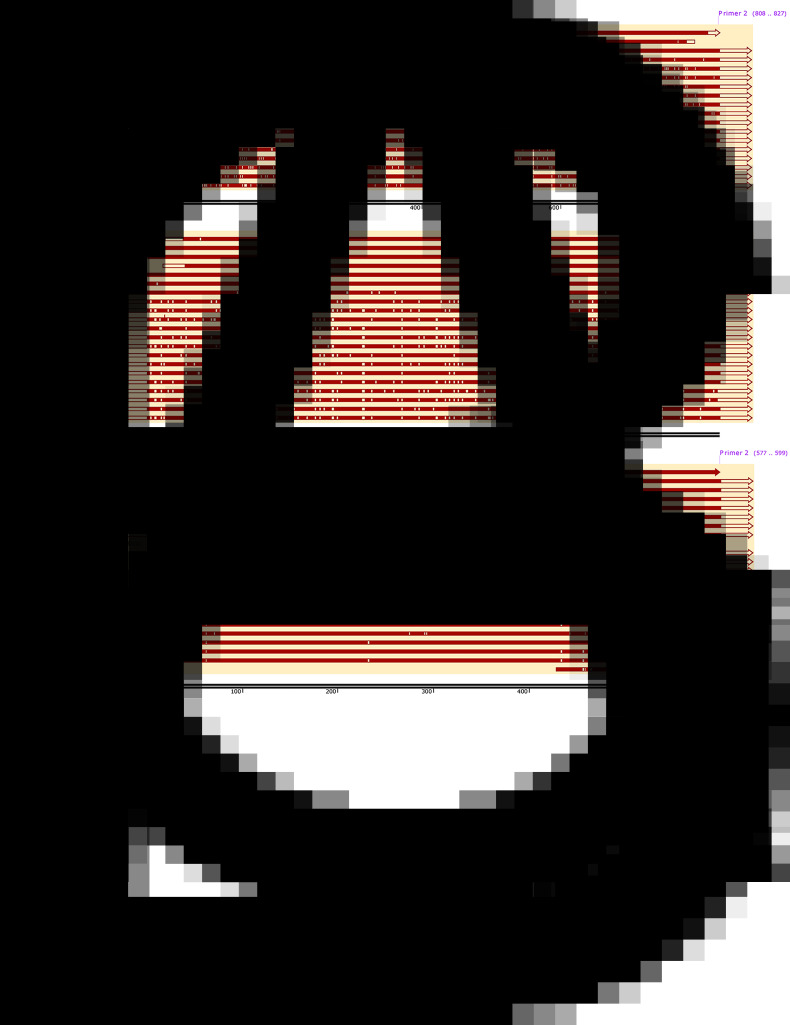
Sequence alignment analysis of (A) COX1 based on Tsag_COX1/F and Tae_COX1/R primers, (B) COX1 based on JB3 and JB4.5 primers and (C) HDP2 sequence.

**Fig. 5.**
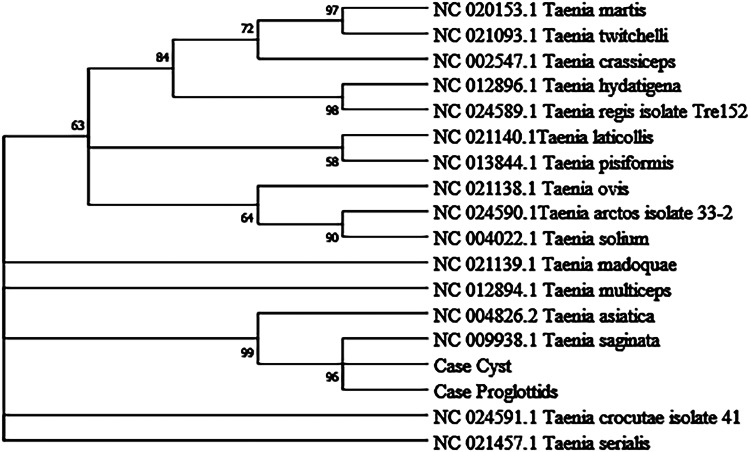
Phylogenetic tree was constructed based on the COX1 sequences of *Taenia* spp. using the maximum-likelihood method.

The proglottid from the patient was successfully amplified and sequenced using 2 pairs of the primers of the target *COX1*. The cyst was amplified with JB3 and JB4.5 primers, and the nuclear DNA HDP2 target fragment was also effectively amplified and sequenced. The 827-bp amplified products of proglottid using the *T. saginata* species-specific forward primer Tsag_COX1/F and *Taenia* common reverse primer Tae_COX1/R were 99.27–100% identical to *T. saginata* and 96.25–96.37% equivalent to *T. asiatica* without other species (Fig. 4). BLAST results indicated that the per cent identity of the 396-bp PCR products of the proglottid and cyst samples, which had trimmed JB3 and JB4.5 primer sequences, was 99.24–100% identical with *T. saginata* (Fig. 4). However, the 599 bp of the HDP2 target sequence revealed 99.67–99.83% identity with *T. saginata*, 95.66–99.67% with *T. asiatica* and 92.99–97.33% with *T. solium* under the same BLAST conditions (Fig. 4). Additional information regarding sequence alignment is provided in the Supplementary material. Based on the *COX1* phylogenetic analysis (Fig. 5), the case is most likely *T. saginata*, next *T. asiatica* and finally *T. solium*.

## Discussion

Taeniasis coexisting with cysticercosis in humans is uncommon, especially for *T. saginata* infection. New insights into the pathogenicity and life cycle of *T. saginata* may be drawn from the case of the Tibetan patient with *Taenia* intestinal infection and cysticercosis (brain, muscles) reported in this paper. The life cycle of *Taenia*, involving the pathogenic mechanism of *Taenia* infection, is significant for ascertaining the disease classification (taeniasis or cysticercosis), epidemiological context, disease control and prevention (Garcia *et al*., 2020). Current medical evidence confirmed the proven diagnosis of human taeniasis caused by *T. saginata* and suggested the diagnosis of muscular cysticercosis and neurocysticercosis caused by the larval stage of uncertain *Taenia* species, which may belong to *T. saginata*, *T. asiatica* or hybrids of the 2 sibling species (Okamoto *et al*., 2010; Yamane *et al*., 2012). Studies have also reported that rare *Taenia* species (*Taenia crassiceps*, *Taenia serialis* and *Taenia martis*) also cause human cysticercosis, confirmed by molecular diagnostic tests (Tappe *et al*., 2016; Mueller *et al*., 2020). The potential pathogenetic mechanism of the larval stage of these species might be similar to *T. solium*, wherein embryos hatch in the small intestine and then may invade and spread haematogenously to the brain or muscle and other tissues (Garcia *et al*., 2020).

Three common *Taenia* species can be differentiated by examination of their morphological characteristics, such as the scolex, mature and gravid proglottids in the adult stage, and the scolex in the larval stage (Eom *et al*., 2020). However, the differential diagnosis between them is challenging when their morphology is not so typically visible. As far as morphological characteristics were concerned, the gravid proglottids (Fig. 3) from this patient were the same as *T. saginata* or *T. asiatica*, not *T. solium*, while the morphological evaluation of cysticercosis, that showed the presence of detached hooks (Fig. 3), referred to *T. asiatica* and *T. solium* rather than *T. saginata*. However, the traditional morphological taxonomy has some inherent limitations leading to possible false-species identification and can neglect cryptic or pseudocryptic species. That is the reason why integrated taxonomy approaches, wherein molecular studies combine detailed morphological information, are important in helping characterize pairing at the individual level resulting in the perfect characterization of cryptic biodiversity (Hebert *et al*., 2003; Laakmann *et al*., 2020).

The most common method for molecular identification of *Taenia* tapeworms has been PCR coupled with the sequencing of the amplified PCR product (Ale *et al*., 2014). Other assays for the differential diagnosis of *Taenia* include multiplex PCR (Nunes *et al*., 2003; Yamasaki *et al*., 2004; Jeon *et al*., 2009; Ng-Nguyen *et al*., 2017), PCR coupled with restriction fragment length polymorphism analysis (Gonzalez et al., 2004), random-amplified polymorphic DNA analysis (Jeon et al., 2009), loop-mediated isothermal amplification (Nkouawa *et al*., 2009) and matrix-assisted laser desorption ionization-time-of-flight mass spectrometry (Wendel *et al*., 2021). Different target sequences of *Taenia* genomic DNA, including mitochondrial DNA (e.g. COX1, CYTb, valine tRNA, NADH, 12S rDNA gene) (Yamasaki *et al*., 2002; Okamoto *et al*., 2010; Roelfsema *et al*., 2016), ribosomal DNA (i.e. 28S rRNA, 5.8S rRNA and ITS rRNA gene) (von Nickisch-Rosenegk *et al*., 1999; Praet *et al*., 2013) and nuclear DNA (HDP1, HDP2, cathepsin L-like cysteine peptidase, *Ag2* gene) (Gonzalez *et al*., 2000; Gonzalez *et al*., 2004; Flores *et al*., 2018), have been used as markers in molecular diagnosis. In particular, the mitochondrial *COX1* gene that has been used in the study is a universally accepted DNA barcoding marker for assessing the genetic variation and evolutionary biology of Metazoa, including helminth parasites (Hebert *et al*., 2003; Galimberti *et al*., 2012; Zhang *et al*., 2014; Rostami *et al*., 2015; Mioduchowska *et al*., 2018; Eberle *et al*., 2020). Molecular characterization in our study based on COX1 and HDP2 indicated that the cyst seemed to be more inclined to *T. saginata* or *T. asiatica*. The analysis of HDP2 sequences in cysticercus samples ruled out the possibility of *T. solium*, but failed to reliably differentiate *T. saginata* from *T. asiatica* or their hybrids (Okamoto *et al*., 2010). High homologies were found between *T. saginata* and *T. asiatica* on mitochondrial *COX1* gene, as shown by the phylogenetic dendrogram (Fig. 5) which is in line with a prior study showing a 4.6% difference between the whole mitochondrial genome sequence of *T. saginata* and *T. asiatica* (Jeon *et al*., 2007). However, the BLAST results of the cyst were inconsistent with its morphology, which encompassed dropping hooks in the slide (Fig. 3). These disagreements in morphology and molecular identification suggested the possibility of hybrids of *T. saginata* and *T. asiatica.*

For studies involving DNA sequences, whole-genome sequencing has become the standard method for collecting relevant data (Eberle *et al*., 2020). Unfortunately, due to the scarcity of high-quality cyst samples, we have not been able to sequence the entire cyst genome. However, our study suggested that patients with taeniasis accompanied by cysticercosis may need whole-genome analysis to further ascertain whether *T. saginata* is also responsible for causing human cysticercosis. Despite the widespread availability of molecular identification methods, often tested on proglottids' samples, the sensitivity of detection in cyst samples may be lowered due to DNA degradation within cysts (Figueiredo *et al*., 2019). Many researchers have reported various mitochondrial/nuclear gene discordances in specimens, indicating potential hybrids between *T. asiatica* and *T. saginata* (Nkouawa *et al*., 2009; Okamoto *et al*., 2010; Yamane *et al*., 2012; Ale *et al*., 2014). Therefore, a molecular sequence-based species characterization that relies on information encoded by a single gene of the mitochondrial genome can be deceptive. Hence, mitochondrial, ribosomal or nuclear marker-based multilocus sequence analysis of genetic heterogeneity within the *Taenia* spp. could be the more effective routine molecular identification in clinical laboratories (Pajuelo *et al*., 2015). Therefore, it would be interesting to develop an easy, simple-to-handle and highly sensitive, multilocus sequences-based molecular diagnostic method for specimen identification of *Taenia* for validation in clinical settings.

In conclusion, the cause of human cysticercosis by non-solium *Taenia* species, such as *T. saginata* or *T. asiatica*, remains unclear. Correct diagnosis can be aided by utilizing whole-genome analysis and molecular identification approaches that focus on multilocus sequences. In order to better understand the comprehensive epidemiology and total life cycle of *Taenia*, the limitations of the existing molecular diagnostic applications require more research for the correct diagnosis and prevention of the disease.

## Data Availability

The data and materials information used and analyzed in the current study are available from the first author (Jie Hou) or corresponding author (Ying Ma) upon reasonable request.

## Supplementary material

The supplementary material for this article can be found at https://doi.org/10.1017/S003118202200169X
